# Analytical validation of the Onco*type* DX prostate cancer assay – a clinical RT-PCR assay optimized for prostate needle biopsies

**DOI:** 10.1186/1471-2164-14-690

**Published:** 2013-10-08

**Authors:** Dejan Knezevic, Audrey D Goddard, Nisha Natraj, Diana B Cherbavaz, Kim M Clark-Langone, Jay Snable, Drew Watson, Sara M Falzarano, Cristina Magi-Galluzzi, Eric A Klein, Christopher Quale

**Affiliations:** 1 Genomic Health, Inc., Redwood City, CA 94063, USA; 2 Robert Tomsich Pathology & Laboratory Medicine Institute, Cleveland Clinic, Cleveland OH, 44195, USA; 3 Glickman Urological and Kidney Institute, Cleveland Clinic, Cleveland, OH 44195, USA

**Keywords:** Prostate cancer, Quantitative RT-PCR, Needle biopsy, Prognostic biomarkers, Analytical validation

## Abstract

**Background:**

The Onco*type* DX® Prostate Cancer Assay is a multi-gene RT-PCR expression assay that was developed for use with fixed paraffin-embedded (FPE) diagnostic prostate needle biopsies containing as little as 1 mm of prostate tumor in the greatest dimension. The assay measures expression of 12 cancer-related genes representing four biological pathways and 5 reference genes which are algorithmically combined to calculate the Genomic Prostate Score (GPS). This biopsy-based assay has been analytically and subsequently clinically validated as a predictor of aggressive prostate cancer. The aim of this study was to validate the analytical performance of the Onco*type* DX Prostate Cancer Assay using predefined acceptance criteria.

**Results:**

The lowest quartile of RNA yields from prostate needle biopsies (six 5 μm sections) was between 19 and 34 ng. Analytical validation of the process requiring as little as 5 ng of RNA met all pre-defined acceptance criteria. Amplification efficiencies, analytical sensitivity, and accuracy of gene assays were measured by serially diluting an RNA sample and analyzing features of the linear regression between RNA expression measured by the crossing point (Cp) versus the log_2_ of the RNA input per PCR assay well. Gene assays were shown to accurately measure expression over a wide range of inputs (from as low as 0.005 ng to 320 ng). Analytical accuracy was excellent with average biases at qPCR inputs representative of patient samples <9.7% across all assays while amplification efficiencies were within ±6% of the median. Assessments of reproducibility and precision were performed by testing 10 prostate cancer RNA samples over multiple instruments, reagent lots, operators, days (precision), and RNA input levels (reproducibility) using appropriately parameterized linear mixed models. The standard deviations for analytical precision and reproducibility were 1.86 and 2.11 GPS units (100-unit scale) respectively**.**

**Conclusions:**

The Onco*type* DX Prostate Cancer Assay, a clinical RT-PCR assay specifically designed for use with prostate needle biopsies, has been analytically validated using very limited RNA inputs. The assay requirements and analytical performance will provide physicians with test results from a robust and reliable assay which will enable improved treatment decisions for men diagnosed with early-stage prostate cancer.

## Background

Prostate cancer is one of the most common malignancies in men with an estimated 250,000 new cases being diagnosed in the US in 2012, mostly as a consequence of PSA screening [1]. Given that most newly diagnosed cases represent low-risk disease, with less that 3% of men dying of prostate cancer, active surveillance rather than immediate treatment has been broadly endorsed by practice guidelines to be a valid treatment option for many men. Nonetheless, a large majority of men with low-risk, early stage disease still undergo aggressive intervention with radical prostatectomy and/or radiation therapy [2], despite their attendant long-term side effects and cost, in large part because clinicians are uncertain as to the accuracy of conventional methods for discriminating low-risk from high-risk disease. A number of risk assessment tools (e.g. nomograms), based on clinical and pathologic features such as serum PSA level, clinical stage and biopsy Gleason Score, are currently being used for risk stratification of men with early-stage prostate cancer [3]. Although these tools have some predictive value, a substantial fraction of men expected to have low-risk disease are found to have more aggressive disease at prostatectomy [4]. Clearly there is a need for more accurate discrimination of low risk disease from aggressive prostate cancers at the time of diagnosis.

There is a growing recognition that molecular biomarkers can complement conventional clinical and pathologic parameters to personalize the care of cancer patients. However, incorporation of biomarkers into standard clinical practice requires a level of validation which is not often achieved. In order for a biomarker to enter wide clinical practice it needs to demonstrate evidence of strong analytical validity, clinical validity, and clinical utility [5]. Indeed, successful clinical validation may be challenging unless the biomarker is based on a robust, analytically validated platform. Genomic Health, Inc. has developed a family of analytically [6,7] and clinically-validated, multi-gene real-time polymerase chain reaction (RT-PCR) assays (Onco*type* DX® Assays) which identify underlying biology in an individual patient’s tumor to help guide treatment decisions in invasive node negative and node positive breast cancer [8-11], breast ductal carcinoma *in situ*[12] and stage II/III colon cancer [13-15]. Since its introduction in 2004, the Onco*type* DX Breast Cancer Assay has become widely used in standard clinical practice, and is incorporated into the major oncology practice guidelines, including NCCN and ASCO [16,17].

In order to impact clinical decision-making at the time of diagnosis, the Onco*type* DX Prostate Cancer Assay was specifically designed for analysis of fixed, paraffin-embedded (FPE) prostate needle biopsy tissue. Some of the key challenges in developing this biopsy-based assay for prostate cancer include the heterogeneous and multifocal nature of the disease, and the very small amounts of tumor tissue available from diagnostic prostate needle biopsies. Most newly diagnosed prostate cancer patients harbor low-volume disease that may be diagnosed from a single biopsy core [18]. A series of analytical studies were performed to optimize sensitivity of the analytical component of the Onco*type* DX Prostate Cancer Assay platform and enable processing of small volume tissue from prostate biopsies. Some of the most notable changes include addition of a multiplexed preamplification step after reverse transcription and combining the genomic DNA detection step with the quantitative PCR step. Considering that the RNA yields of many samples cannot be reliably measured with current methods, the sample quality is determined primarily by the expression of reference genes rather than mass as a measure of the amplifiable RNA. The analytical portion of the Onco*type* DX Prostate Cancer Assay was designed to target 20 ng as the nominal input, however the assay accommodates samples with lower concentration through variable RNA inputs, thus enabling the assay to perform well with RNA inputs which are 110–180 fold lower than required by the Onco*type* DX Colon and Breast Cancer Assays [6,7]. The Onco*type* DX Prostate Cancer Assay includes 5 reference genes and 12 cancer genes representing distinct biological pathways with a known role in prostate tumorigenesis: the androgen pathway (*AZGP1, KLK2, SRD5A2*, and *FAM13C*), cellular organization (*FLNC, GSN, TPM2,* and *GSTM2*), proliferation (*TPX2*), and stromal response (*BGN, COL1A1,* and *SFRP4*). Reference gene normalization is used to control for sources of pre-analytical and analytical variability as well as allow for variable RNA inputs. Reference normalized expression of the 12 cancer-related genes are used to calculate the Genomic Prostate Score (GPS), which has been shown to predict adverse prostate cancer pathology beyond conventional clinical/pathologic factors in a recently completed clinical validation study [19].

Prior to initiation of the clinical validation study, analytical validation studies, with pre-specified endpoints and acceptance criteria, were conducted to ensure that the analytical component of the Onco*type* DX Prostate Cancer Assay is well-controlled and produces reliable assessment of RNA from individual FPE prostate tumor tissue. These studies demonstrated analytical validity of the gene assays comprising the Onco*type* DX Prostate Cancer Assay with respect to analytical sensitivity, bias, amplification efficiency, precision and reproducibility, and also validated the precision and reproducibility of the resulting GPS.

## Methods

### Tumor blocks and samples

FPE needle biopsies were provided by the Cleveland Clinic Pathology and Laboratory Medicine Institute and Glickman Urological and Kidney Institute (C. M-G., E.A.K., S.M.F.) and were centrally reviewed by two pathologists (C.M-G and S.M.F). The use of the samples has been approved by the Cleveland Clinic Institutional Review Board. All biopsies were sectioned into eight 5 μm sections. The top and the bottom slides were hematoxylin and eosin (H&E) stained and the presence of tumor was confirmed by a board-certified pathologist. The tumor area was marked in the top H&E slide and the markings were transferred to the subsequent 6 unstained slides. The tumor was manually dissected from each unstained slide using a scalpel blade and transferred into a microcentrifuge tube. FPE prostate cancer samples from radical prostatectomies were purchased from ProteoGenex (Culver City, CA). The needle biopsy tumors were microdissected to exclude normal-appearing tissue. The selected radical prostatectomy blocks spanned a wide range of gene expression and range of GPS. Gleason score was assigned using the 2005 International Society of Urological Pathology Consensus guidelines [20].

### RNA extraction

Paraffin from FPE samples was solubilized by Shandon Xylene Substitute (Thermo Fisher Scientific, Waltham, MA) and removed. Tissue was lysed and protein was digested with Proteinase K (800μg; Beckman Coulter, Beverly, MA). Nucleic acids were bound to paramagnetic beads using the Agencourt® FormaPure Kit (Beckman Coulter, Beverly, MA) and manipulated using Tecan® liquid handling robots (Tecan, Männedorf, Switzerland) with an integrated KingFisher® Flex magnetic particle processor (Thermo Fisher Scientific, Waltham, MA). DNA was digested with 200 units of DNaseI (Promega, Madison, WI). Purified RNA was released from the paramagnetic beads and suspended in water.

### RNA quantitation

RNA was quantified using the RiboGreen® fluorescence method (Invitrogen, Carlsbad, CA) according to the manufacturer’s instructions. The limit of quantitation for the assay was 0.5 ng/μL.

### Reverse transcription

RNA (up to 20 ng) was converted to complementary DNA (cDNA) by combining the Omniscript® RT kit (Qiagen, Valencia, CA) and a specific reverse primer for each gene assay using Tecan liquid handling robots. All primers (final concentration 50 nmol/L) were purchased from Integrated DNA Technologies (Coralville, IA). The RT reaction (30 μL) was incubated at 37°C for 60 minutes and then inactivated at 93°C for 5 minutes.

### Preamplification

The cDNA was preamplified using custom TaqMan® PreAmp Master Mix (Life Technologies, Carlsbad, CA) and custom forward and reverse primers (55 nM final concentration) for each target gene, including the control assay for genomic DNA (gDNA) detection. The reactions were assembled using Tecan liquid handling robots, placed in a thermocycler (BioRad, Hercules, CA) and incubated under the following conditions: 95°C for 10 min followed by 8 cycles of 95°C for 15 sec and 60°C for 4 min.

### qPCR and genomic DNA detection

The amplified product was mixed with the forward and reverse primers and probes (Black Hole Quencher-2, Integrated DNA Technologies) for each of the gene assays and for the gDNA detection assay (*ARF1_promoter*, designed to amplify a promoter region of *ARF1*) using Tecan liquid handling robots. QuantiTect® Primer Assay master mix (Qiagen, Valencia, CA) was used, and the 5 μL reaction was amplified for 45 cycles in a LightCycler® 480 (Roche Applied Science, Indianapolis, IN) under the following conditions: enzyme activation (95°C, 15 min), amplification (95°C for 20 sec and 60°C for 45 sec; 45 cycles in total), cooling (40°C, 5 sec). The level of expression was calculated with the crossing point (Cp) method implemented by the Roche LightCycler 480 software version 1.5. All gene assays were measured in triplicate and required at least 2 valid wells. Oligonucleotide and amplicon sequences can be found in the Additional file 1 and Additional file 2, respectively.

### Reference gene normalization and Genomic Prostate Score calculation

**Figure 1:**
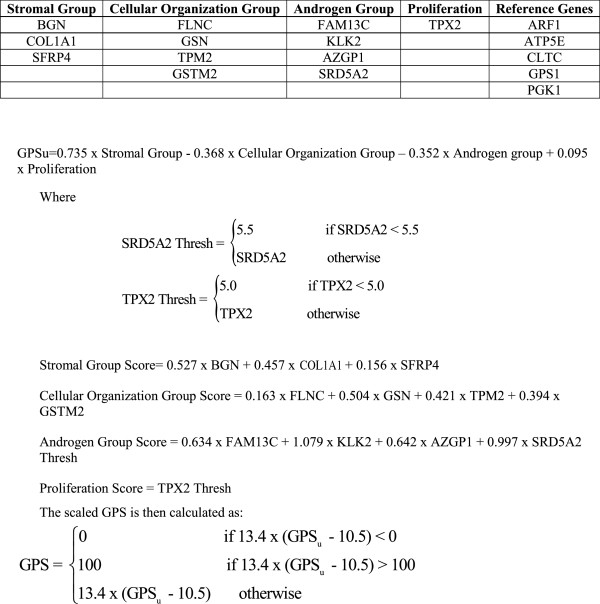
**Calculation of Genomic Prostate Score (GPS).** The aggregate expression of 5 reference genes was used to reference normalize the expression of the 12 cancer-related genes. Normalized gene expression was used to calculate the individual group scores: stromal group score, cellular organization score, androgen groups score and proliferation score. Each of those group scores is algorithmically combined to calculate the unscaled Genomic Prostate Score (GPSu); the GPSu is then scaled to a 100-unit range GPS. A negative coefficient in the calculation of the GPS is associated with better outcome whereas a positive coefficient is associated with poorer outcome.

The five reference genes (*ARF1, ATP5E, CLTC, GPS1* and *PGK1*) were selected in an independent study for their low inter-patient variability, lack of relationship to clinical outcome and robust analytical performance (data not shown). Reference gene normalization was used to adjust for potential sources of pre-analytical variability, such as fixation, RNA fragmentation and tissue quality, analytical variation including assay plate (RT, qPCR) and instrumentation (liquid handler or LC480), and varying RNA inputs (with 20 ng as the target input). Gene expression was normalized by subtracting the aggregated expression of the reference genes from the weighted mean Cp for each of the 12 informative genes and centering by adding 10 units to the result. The formula used to calculate the GPS is shown in Figure 1.

### Linearity

Linearity was assessed by serially diluting RNA and analyzing gene expression as a function of 14 input concentrations of RNA extracted from FPE prostate tumor tissue. Two different starting RNA concentrations were used (1,638 ng/well and 204 ng/well) where the higher starting concentration was used for the genes with the lowest expression levels (*TPX2* and *SRD5A2*), and the lower starting concentration was used for all other genes. For each starting concentration, three independent series of two-fold serial dilutions were prepared using a Tecan liquid handling robot. The serially diluted RNA was converted to cDNA, preamplified for 8 cycles of PCR and taken through 45 cycles of qPCR. The linearity of signal response between the measured gene expression (Cp) and the log base 2 of RNA concentration was evaluated for each of the individual gene assays. In total, for each gene and RNA concentration, 9-replicate PCR measurements were recorded and analyzed.

Linearity was assessed for each gene using the polynomial method developed by Krouwer *et al*. [21]. The polynomial method utilized in these analyses first fit a quadratic and cubic regression models and compares, through tests of significance on the quadratic and/or cubic terms, whether the extra linear models are better fits to the data than a linear regression. Heteroscedasticity of the error variance was modeled as a log-linear function of (known) log input concentration. These tests were used to determine the presence of significant non-linearity (at a 0.05 alpha level). If significant non-linearity was detected, then the predicted Cp value from the best fitting polynomial model was compared with the corresponding predicted Cp value from the linear model. This difference in predicted Cp values (deviation from linearity, (DL)) was calculated at each input concentration. A maximum DL across concentrations of < 1 Cp was used to determine that a gene is sufficiently linear over the concentrations studied. For a given concentration, if the DL was greater than or equal to 1 Cp, then that concentration was removed from the linear range until all concentrations met the DL criteria, thereby defining the limit of quantitation (LOQ). Additionally, to control large residual variation at low concentrations, which is a consequence of preamplification of small starting amounts of cDNA, an additional criterion was applied where concentrations below and including the point at which the residual standard deviation was > 1.5 Cp were removed from the linear range, and polynomial model assessments were repeated.

### Amplification efficiency

Estimates of amplification efficiencies were obtained for all gene assays and are provided by the formula:

$$\mathrm{Efficiency}=100\times \left(2^{-1/\mathrm{slope}}-1\right)$$

where the slope is estimated from the regression of Cp measurements versus (known) log base 2 RNA concentration described above.

### Analytical accuracy

The analytical accuracy of predicted RNA concentrations relative to the (known) input RNA concentrations was estimated for each of the 17 genes separately. Specifically, for every evaluable Cp measurement, an inverse prediction of RNA concentration was derived from the final linear model:

$$y={\hat{b}}_{0}+{\hat{b}}_{1}\log_{2}\left(x\right)$$

An estimate of the accuracy of the assay was given by the mean percent bias in prediction at each known RNA concentration *k* (*k* = 1…14); namely, by

$${\bar{\text{Bias}}}_{k}=\frac{\sum_{i=1}^{3}\sum_{j=1}^{m_{i}}{\hat{x}}_{\mathrm{ijk}}-x_{k}}{mx_{k}}$$

where ^*X*^_*k*_ is the (known) input RNA concentration for the k^th^ level and ${\hat{x}}_{\mathrm{ijk}}$ was the predicted RNA concentration obtained from inverse prediction for the i^th^ plate (i=1,2,3), j^th^ well (j=1,2,3) and k^th^ RNA concentration level, *m*_*i*_ was the number of valid wells on the i^th^ plate, and *m* was the total number of valid wells across all plates. Analyses were performed using restricted maximum likelihood estimation (REML) in the PROC MIXED procedure of SAS version 9.2.

### Analytical sensitivity

The analytical sensitivity of each of the gene assays was evaluated separately. Specifically, for each gene a nonlinear mixed effects model with log-linear variance function or unstructured covariance was used to model the expected heteroscedasticity in intra-assay response as a function of RNA concentration. This information was used to estimate the Limit of Detection (LOD) and LOQ of the assay on the Cp scale. For each of the 17 gene assays, the LOD was estimated by a lower 95% confidence bound on the mean expression at zero concentration (natural original scale). Similarly, the LOQ on the Cp scale was estimated by the upper one-sided 95% confidence interval of the fitted (linear) model at the inverse prediction of y_min_ or at the minimum concentration fulfilling the linear range criteria if the inverse prediction of y_min_ was less than the lower linear range limit (on the concentration scale). Analyses were performed using an iterative estimation scheme involving the PROC MIXED and the PROC NLMIXED procedure in SAS version 9.2.

### Analytical precision and reproducibility

Data for the precision and reproducibility were generated from 10 different FPE prostate cancer samples that were run 9 times each: 3 replicates at each of 3 different RNA inputs (5 ng, 10 ng, and 20 ng). Both precision and reproducibility were generated using multiple lots of RT-PCR reagents, multiple PCR instruments and multiple Tecan liquid-handling robots. The precision component for each of the 12 informative genes as well as the GPS was defined as the within RNA input standard deviation captured in the residual variation term from the linear mixed model described below:

$$Y_{\mathrm{ijk}}=\mu +\alpha_{i}+\lambda_{j}{+\epsilon }_{\mathrm{ijk}}$$

Where Y_ijk_ was the outcome measurement (either GPS or reference normalized gene expression), μ was the overall mean effect, α_i_ was the random effect of the i^th^ sample (i=1,2,..,10), distributed N(0,σ_α_^2^), λ_j_ was the fixed effect of the j^th^ RNA Input (j=1,2,3), ϵ_ijk_ was the residual error ~ N(0, σ_ϵ_^2^) where k indexes replicate (k=1,2,3), assumed independent of α_i_.

The reproducibility component for each of the 12 informative genes as well as the GPS was defined as the within and between RNA input standard deviation, captured in the residual variation term from the linear mixed model described below:

$$Y_{\mathrm{ijk}}=\mu +\alpha_{i}{+\epsilon }_{\mathrm{ijk}}$$

Where Y_ijk_ was the outcome measurement (either GPS or reference normalized gene expression), μ was the overall mean effect, α_i_ was the random effect of the i^th^ sample (i=1,2,..,10), distributed N(0,σ_α_^2^), ϵ_ijk_ was the residual error ~ N(0,σ_ϵ_^2^) where k indexes replicate (k=1,2,3), assumed independent of α_i_.

### Assay controls

Each Onco*type* DX Prostate Cancer Assay RT and preamplification assay plate has 5 wells containing processing controls: RNA extracted from prostate cancer FPE (a positive control for reverse transcription, preamplification and qPCR), human genomic DNA (a positive control for gDNA detection, Promega, Madison, WI), and 3 wells containing nuclease-free water (a negative control for contamination). In addition, all qPCR plates have 12 wells of positive (gDNA) and 12 wells of negative (nuclease-free water) controls.

## Results

### Distribution of RNA yields in the smallest diagnostic prostate biopsies

**Figure 2:**
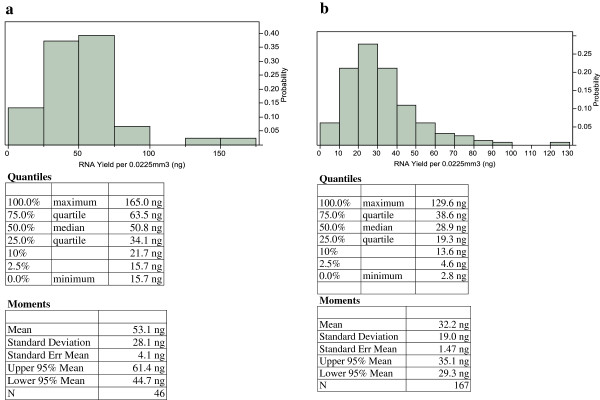
**Estimates of RNA yields in the smallest diagnostic biopsies. (a)** RNA from 46 formalin-fixed prostate needle core biopsy specimens collected between 2003 and 2008 by the Cleveland Clinic was extracted and RNA yields were measured using the RiboGreen method. Tumor volumes were measured and all results were adjusted to represent RNA yields in the target tumor volume of 0.0225 mm^3^ (1 mm tumor length x 0.75 mm width of diagnostic biopsies x 0.030 mm tumor depth). **(b)** AUA low and intermediate risk patients treated with radical prostatectomy at Cleveland Clinic between 1999 and 2007 with available biopsy tissues were included in the study. In total, RNA from 167 diagnostic biopsies was extracted and RNA yields were measured using the RiboGreen method. Tumor volumes were measured and all results were adjusted to represent RNA yields in the target tumor volume of 0.0225 mm^3^ (1 mm tumor length x 0.75 mm width of diagnostic biopsies x 0.030 mm tumor depth).

We estimated that the smallest diagnostic biopsies would contain 0.0225 mm^3^ of tumor volume (1 mm tumor length × 0.75 mm biopsy core width × 0.030 mm tumor depth (based on availability of six 5 μm sections)) and sought to investigate distribution of RNA yields in such samples. In two separate studies [22,23], RNA was extracted from 46 and 167 biopsies. Using this data, the average RNA yields in the targeted tumor volume were estimated to be less than 50 ng and the lowest quartile was estimated to contain between 19 and 34 ng of RNA (Figure 2a and 2b). Considering variability in clinical practice, it is expected that biopsies from men with prostate cancer will be encountered which contain less than this amount of RNA; as such, we targeted an RNA input for the assay below the lower limit of the lowest quartile, in order to meet the needs of all patients.

### Amplification efficiency

In order for normalization and multianalyte GPS result calculation to be accurate, amplification efficiencies of individual gene assays were required to be similar [24], defined as the median ± 20%. The median amplification efficiency for the 17 gene assays was 93.9% and all efficiencies were within ± 6.3% of that value, demonstrating that gene-specific assay performance was comparable for all assays. Amplification efficiencies ranged from 88% (*AZGP1*) to 100% (*TPM2*). Amplification efficiencies of individual assays, including 95% confidence intervals are shown in Table 1.

**Table 1:** **Amplification efficiencies of individual gene assays in the Onco *type *** **DX Prostate Cancer Assay reporting the GPS**

**Gene Assay**	**Estimated Amplification Efficiency**	**Lower 95% CI**	**Upper 95% CI**
ARF1	93.9	92.8	95.0
ATP5E	90.2	88.2	92.2
AZGP1	88.1	86.5	89.7
BGN	92.5	90.5	94.7
CLTC	97.2	95.9	98.6
COL1A1	95.4	94.5	96.3
FAM13C1	88.5	85.8	91.3
FLNC	96.9	94.9	99.0
GPS1	94.4	92.4	96.6
GSN	91.5	89.7	93.4
GSTM2	95.9	93.8	98.2
KLK2	92.4	91.7	93.1
PGK1	89.9	88.4	91.4
SFRP4	93.9	91.2	96.7
SRD5A2	98.7	96.3	101.4
TPM2	100.2	98.8	101.8
TPX2	98.9	97.1	100.7

Estimated amplification efficiencies and 95% confidence intervals for the 17 genes comprising the GPS derived from a 15-point dilution series.

### Analytical sensitivity

For a multianalyte assay to be clinically useful, it must also be able to distinguish between signal and background non-specific signal. All gene assays had LODs that surpassed the pre-specified Cp criteria of 35. Moreover, to make accurate expression measurements, LOQs need to be in a range of Cp values that are higher than the estimates for gene expression within the intended specimen type. Acceptance criteria were pre-specified for each gene assay separately, and all were met. Most of the assays had estimated LOQs between Cp=33 and Cp=35 effectively indicating that most individual assays can accurately quantitate several copies of RNA. The lowest estimated LOQ of 29.4 was for the *KLK2* assay. Considering that this value was established at its final dilution point, it is possible that the true LOQ is higher. However, even with this estimated LOQ, the discriminative capability of the *KLK2* assay is appropriate for the expected population distribution (median population expression level has been estimated to be approximately Cp=20). Table 2 shows data for the estimated LOQ and LOD for each gene assay.

**Table 2:** Limits of quantitation and detection

**Gene Assay**	**Est. LOQ (Cp)**	**Est. LOD (Cp)**
ARF1	33.4	38.9
ATP5E	33.6	40.0
AZGP1	34.7	40.0
BGN	35.3	36.3
CLTC	34.3	40.0
COL1A1	33.2	37.0
FAM13C1	35.3	40.0
FLNC	32.7	40.0
GPS1	33.5	40.0
GSN	33.4	40.0
GSTM2	33.2	40.0
KLK2	29.4	40.0
PGK1	35.4	40.0
SFRP4	34.8	40.0
SRD5A2	33.5	36.6
TPM2	33.4	40.0
TPX2	34.4	36.3

Analytical sensitivity (estimated Limits of Quantitation and Limits of Detection) for each of the 17 genes comprising the GPS derived from the 15-point dilution series.

### Linear range

FPE RNA was serially diluted two-fold and performance of the individual gene assays was assessed over a linear range of 14 RNA concentrations. Table 3 lists the linear ranges for all gene assays, which ranged from 10 log units (*FLNC, GSTM2*) to the full 14 log units (16,384-fold) RNA concentrations studied (*ARF1, BGN, CLTC, COL1A1, KLK2, TPM2*). The smallest amounts that could be accurately quantified ranged from 0.025 ng to 1.6 ng RNA per PCR well. These results demonstrate the performance of these gene assays to quantify cDNA over a wide range of input concentrations that can be encountered in small prostate needle biopsies.

**Table 3:** Linear range

**Gene Assay**	**Linear Range (ng/well)**	**Quadratic p-value**	**Cubic p-value**	**Maximum Absolute Deviation from Linearity**
ARF1	2^-5.3^ to 2^7.7^	<.0001	0.1191	0.37
ATP5E	2^-3.3^ to 2^7.7^	<.0001	0.0418	0.82
AZGP1	2^-3.3^ to 2^7.7^	0.0006	0.1143	0.46
BGN	2^-5.3^ to 2^7.7^	0.0030	0.4064	0.57
CLTC	2^-5.3^ to 2^7.7^	0.0145	0.2198	0.28
COL1A1	2^-5.3^ to 2^7.7^	0.2406	0.0268	0.31
FAM13C1	2^-3.3^ to 2^7.7^	0.9180	0.0026	0.81
FLNC	2^-1.3^ to 2^7.7^	0.0002	0.0001	0.17
GPS1	2^-3.3^ to 2^7.7^	0.3704	<.0001	0.40
GSN	2^-2.3^ to 2^7.7^	<.0001	0.8081	0.57
GSTM2	2^-1.3^ to 2^7.7^	0.2461	0.0009	0.23
KLK2	2^-5.3^ to 2^7.7^	0.3600	0.0008	0.16
PGK1	2^-3.3^ to 2^7.7^	0.0224	0.5250	0.30
SFRP4	2^-2.3^ to 2^7.7^	0.5033	0.1019	N/A
SRD5A2	2^0.7^ to 2^10.7^	0.0002	0.1016	0.34
TPM2	2^-5.3^ to 2^7.7^	<.0001	0.2190	0.43
TPX2	2^0.7^ to 2^10.7^	0.5396	0.5799	N/A

N/A: No statistically significant departure from linearity.

Estimated linear ranges, associated p-values for quadratic or cubic regression models and maximum deviations from linearity for each of the 17 genes. The results were calculated using the data obtained from the 15-point dilution series.

### Analytical accuracy

For all 17 GPS gene-specific assays, the absolute value of the average percent accuracy at the nominal, one-half and the one-quarter qPCR input was required to be less than 25% in order to meet pre-specified criteria. All assays met these criteria and the results for accuracy are listed in Table 4. The largest analytical bias was observed for the *ATP5E* assay (9.7%) and all other assays displayed a bias smaller than 5% in absolute value. Both the amplification efficiency and the accuracy at the individual concentrations are measures of the assay’s ability to detect the doubling in the amount of RNA for individual genes in 1 Cp increments. The combination of low measures of biases and amplification efficiencies approaching 100% demonstrated robust analytical performance at RNA inputs expected to be found in prostate biopsies.

**Table 4:** Analytical accuracy

**Gene Assay**	**Average % Bias**
ARF1	3.0
ATP5E	9.7
AZGP1	1.8
BGN	3.6
CLTC	−0.3
COL1A1	1.1
FAM13C1	−0.8
FLNC	1.2
GPS1	4.9
GSN	4.8
GSTM2	3.1
KLK2	2.2
PGK1	1.9
SFRP4	3.9
SRD5A2	−1.9
TPM2	1.0
TPX2	−2.0

The estimated analytical accuracy of predicted RNA concentrations relative to the known input RNA concentrations for each of the 17 Onco*type* DX Prostate Cancer Assay gene assays; the average % bias is taken over the nominal, ½ and ¼ qPCR input levels.

### Precision and reproducibility

Precision was evaluated by examining variation within the same RNA input, and reproducibility incorporated both within and between RNA input variation. Pre-specified acceptance criteria were set for the average within RNA input standard deviation pooled between 20 ng, 10 ng and 5 ng RNA input (precision) and the within and between RNA input (20 ng, 10 ng and 5 ng) standard deviation (reproducibility). The lowest input of 5 ng was chosen to mirror the lowest 2.5 percentile of the 0.0225 mm^3^ tumors. A total of 10 blocks were chosen to span a wide range of GPS results. Each RNA extract was processed three separate times at three different input levels (5 ng, 10 ng and 20 ng) by several operators over a two week period. Multiple lots of oligonucleotides, preamplification master mixes, and qPCR master mixes were used and processed on different Tecan liquid-handling robots and LightCycler 480 instruments. The standard deviation for precision was 0.21 Cp or smaller for individual gene assays. For GPS, standard deviation was 1.86 units on the 100-unit scale. The standard deviation for reproducibility was also 0.21 Cp or smaller for all assays. Reproducibility variation for the GPS was 2.11 units on the 100-unit scale. Tables 5 and 6 show results for the gene assays and the GPS for precision and reproducibility, respectively. All precision and reproducibility measurements met pre-specified acceptance criteria thus demonstrating a robust analytical performance. These results establish that average of the reference genes can be used to enable processing of variable RNA inputs while maintaining high precision and reproducibility.

**Table 5:** Analytical precision for normalized gene expression and GPS

**Gene Assay / Score**	**Precision SD reference normalized Cp**	**Lower 95% CL, Cp**	**Upper 95% CL, Cp**
ARF1	0.08	0.07	0.10
ATP5E	0.15	0.13	0.18
AZGP1	0.14	0.12	0.16
BGN	0.11	0.09	0.13
CLTC	0.07	0.06	0.09
COL1A1	0.10	0.08	0.11
FAM13C1	0.10	0.09	0.12
FLNC	0.11	0.09	0.13
GPS1	0.08	0.07	0.09
GSN	0.10	0.09	0.12
GSTM2	0.13	0.11	0.15
KLK2	0.08	0.07	0.09
PGK1	0.08	0.07	0.10
SFRP4	0.11	0.10	0.13
SRD5A2	0.20	0.17	0.23
TPM2	0.10	0.09	0.12
TPX2	0.21	0.18	0.25
**GPS**	**1.86**	**1.60**	**2.20**

Analytical precision (assay variation within a given RNA input level ) of the 17 Onco*type* DX prostate gene assays and GPS was derived from 10 different FPE prostate cancer samples that were run 9 times each: 3 replicates at each of 3 different RNA inputs: 5 ng, 10 ng and 20 ng (target). The within RNA input level sources of variation include instruments (qPCR and Tecan liquid handling robots), reagents lots (oligonucleotides, preamplification master mix and qPCR master mix), operator and time (processing spanned 2 calendar weeks).

**Table 6:** Analytical reproducibility for normalized gene expression and GPS

**Gene Assay / Score**	**Reproducibility SD, reference normalized Cp**	**Lower 95% CL**	**Upper 95% CL**
ARF1	0.09	0.08	0.11
ATP5E	0.17	0.15	0.20
AZGP1	0.15	0.13	0.18
BGN	0.11	0.09	0.13
CLTC	0.07	0.06	0.09
COL1A1	0.10	0.09	0.12
FAM13C1	0.11	0.09	0.12
FLNC	0.11	0.10	0.13
GPS1	0.08	0.07	0.09
GSN	0.14	0.12	0.16
GSTM2	0.14	0.12	0.16
KLK2	0.08	0.07	0.09
PGK1	0.10	0.09	0.12
SFRP4	0.12	0.10	0.14
SRD5A2	0.20	0.17	0.23
TPM2	0.10	0.09	0.12
TPX2	0.21	0.18	0.25
**GPS**	**2.11**	**1.83**	**2.50**

Analytical reproducibility (assay variation incorporating both between RNA input level and within RNA input level variation) of the 17 Onco*type* DX prostate gene assays and GPS was derived from 10 different FPE prostate cancer samples that were run 9 times each: 3 replicates at each of 3 different RNA inputs: 5 ng, 10 ng and 20 ng (target). The within RNA input level sources of variation include instruments (qPCR and Tecan liquid handling robots), reagents lots (oligonucleotides, preamplification master mix and qPCR master mix), operator and time (processing spanned 2 calendar weeks).

### Controls

**Figure 3:**
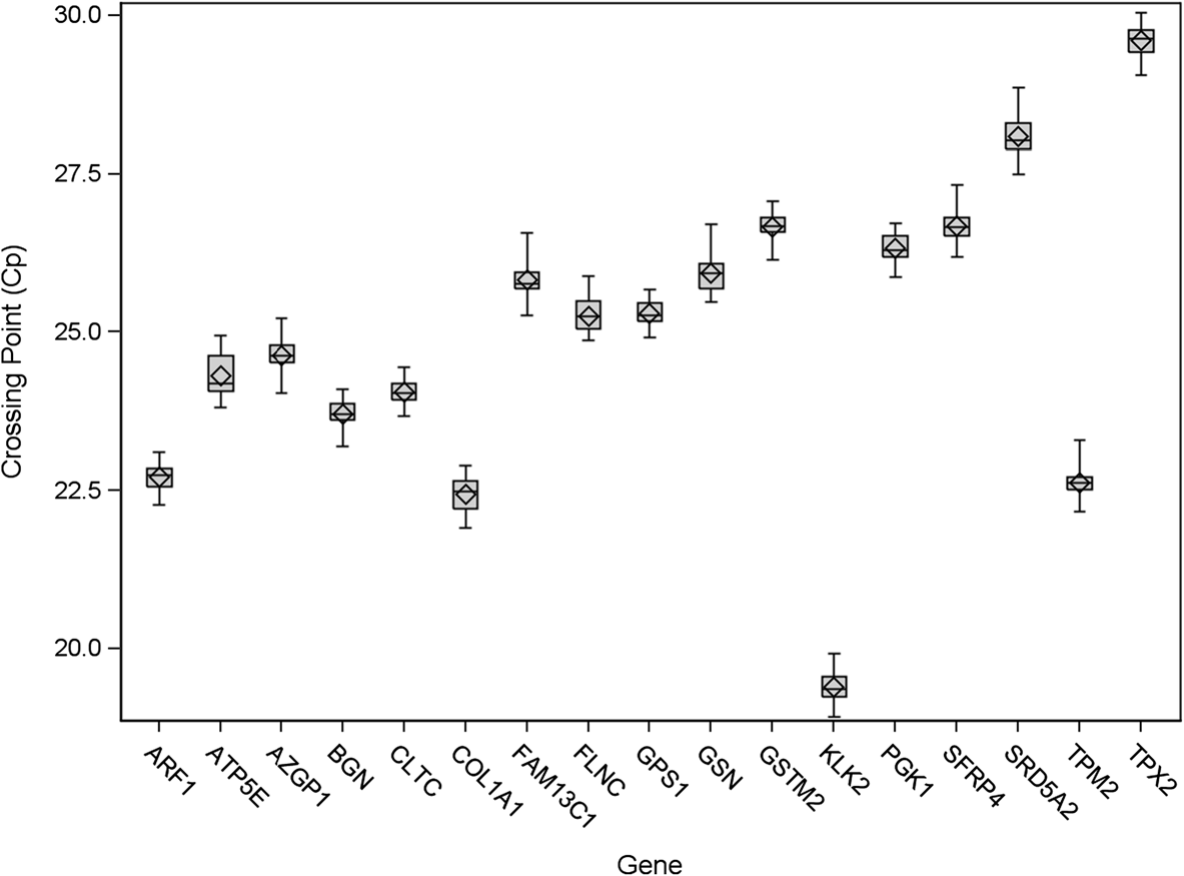
**Boxplots for the RT-PCR positive control during analytical and clinical validation studies.** Boxplots summarizing performance of each gene over analytical and clinical validation for RT-PCR positive control (prostate cancer FPE pool) representing 18 RT-PCR control plates stratified by gene (ARF1, ATP5E, CLTC, GPS1 and PGK1 are reference genes). Each RT-PCR control plate contains one positive control and each gene is measured in triplicate. Standard deviations ranged from 0.19 Cp to 0.33 Cp. The box represents the inter-quartile range, the line in the box represents the median and the diamond is centered at the mean. The whiskers represent minimum and maximum values observed.

**Figure 4:**
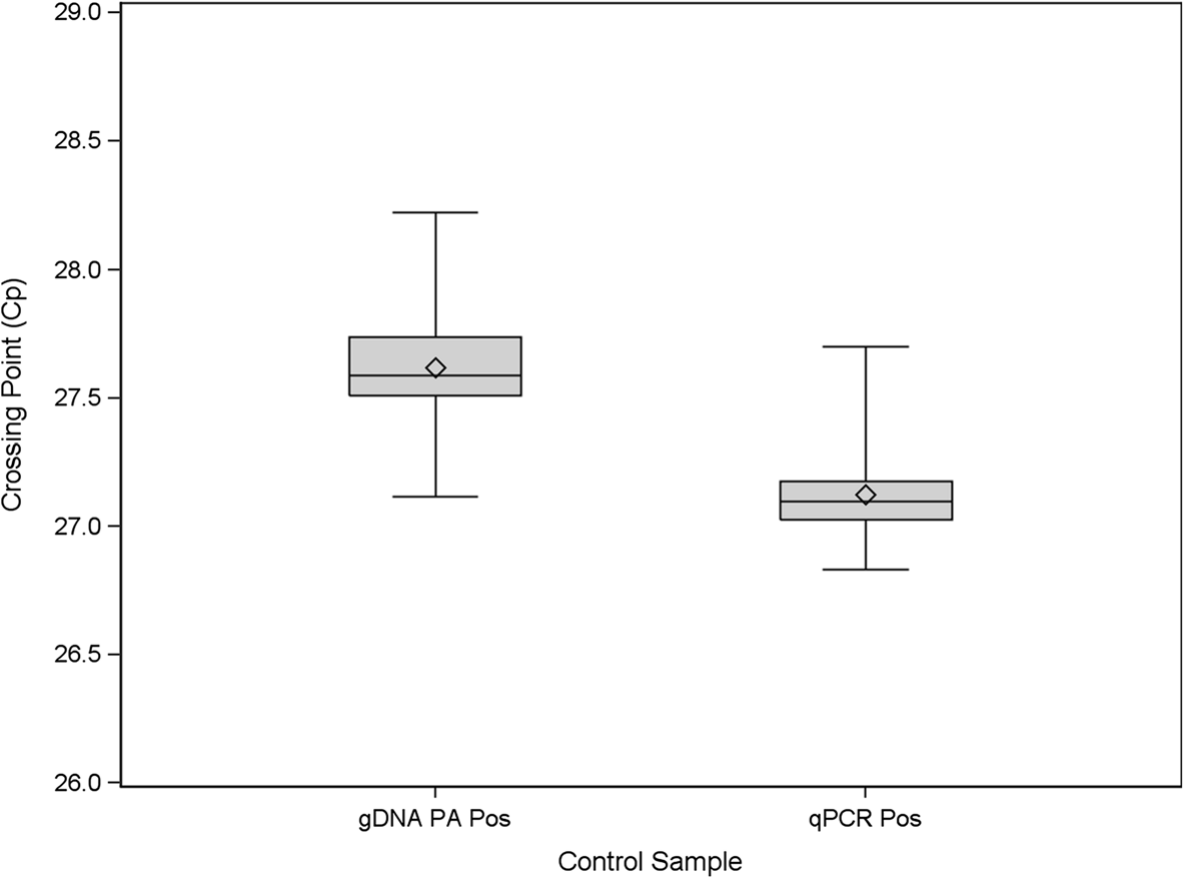
**Boxplots of genomic DNA detection and qPCR positive controls.** Boxplots summarizing the performance of genomic DNA detection and qPCR positive controls over Analytical and Clinical Validation. The box represents the inter-quartile range, the line in the box represents the median and the diamond is centered at the mean. The whiskers represent minimum and maximum values observed.

The box plots presented in Figure 3 illustrate the performance of the RT-PCR positive control across 18 control plates (54 individual Cp measurements per gene) run during analytical validation and the subsequent clinical validation. Table 7 lists the summary statistics, including standard deviations which were calculated on the non-normalized scale. These plates were run on multiple PCR and liquid handling robots by multiple operators, using multiple lots of critical reagents spanning a period of 20 weeks. The performance illustrates a process that is well in control with overall very low variation (the largest SD was 0.33 Cp). Figure 4 presents box plots of the gDNA and qPCR positive control, which are also reflective of good assay performance due to their low variability.

**Table 7:** Performance of the RT-PCR positive control

**Gene Assay**	**Mean**	**Median**	**SD**
ARF1	22.7	22.7	0.20
ATP5E	24.3	24.2	0.33
AZGP1	24.6	24.6	0.23
BGN	23.7	23.7	0.22
CLTC	24.0	24.0	0.19
COL1A1	22.4	22.5	0.25
FAM13C1	25.8	25.8	0.23
FLNC	25.3	25.3	0.24
GPS1	25.3	25.3	0.20
GSN	25.9	25.9	0.29
GSTM2	26.7	26.7	0.21
KLK2	19.4	19.4	0.20
PGK1	26.3	26.3	0.22
SFRP4	26.7	26.7	0.23
SRD5A2	28.1	28.0	0.32
TPM2	22.6	22.6	0.21
TPX2	29.6	29.6	0.25

Summary statistics, including standard deviations for the RT-PCR positive control performance during analytical and clinical validation were calculated on the non-normalized scale. The plates were assembled using multiple PCR and liquid handling robots and by multiple operators, using multiple lots of critical reagents spanning a period of 20 weeks (N=54 individual Cp measurements).

## Discussion

Prostate cancer is one of the most common malignancies in adult males and is typically diagnosed using small needle core biopsies. While many men with newly diagnosed prostate cancer harbor indolent disease, most are treated with immediate surgery or radiation. A molecular assay capable of obtaining reliable, clinically relevant genomic information from small amounts of tumor tissue available in diagnostic core biopsies could help more accurately identify men with low risk of clinical progression who could be managed by active surveillance. In this manuscript we demonstrate that the Onco*type* DX Prostate Cancer Assay can accurately and reproducibly quantitate gene expression at RNA input levels 110–180 fold less [6,7] than the other Onco*type* DX assays.

The Onco*type* DX Prostate Cancer Assay is a multi-gene RT-PCR assay specifically designed to analyze underlying tumor biology in tumor tissue from diagnostic FPE core needle biopsies. In order for a biomarker to enter wide clinical practice, evidence of strong analytical validity needs to be demonstrated [5]. Prior analytical validation studies performed by Genomic Health, Inc. have recently been cited as a model of how such validation studies should be performed [25] and a similar approach to analytical validation was used for the Onco*type* DX Prostate Cancer Assay. All pre-specified analytical criteria (i.e. amplification efficiency, analytical sensitivity, bias, reproducibility and precision) were met, and the analytical assay has been demonstrated to be reliable for clinical use even for patients from whom only limiting amounts of biopsy material are available.

Prostate cancer is often diagnosed from a single positive diagnostic biopsy containing no more than 1 mm of tumor in the greatest dimension. Data suggested that the lowest quartile of such biopsies with 30 microns of available tissue would contain between 19 and 34 ng of RNA. In order to enable accurate and reproducible quantitation of gene expression when limiting copies of target RNA are present, a number of analytical changes to the Onco*type* DX platform used for Breast and Colon cancer were required. More specifically, the multiplexed preamplification step was introduced to create multiple copies of the starting RNA prior to quantitative assessment of gene expression. Given the very low yields and the resulting limitations to accurate measurement of extracted RNA for many samples, acceptance criteria for the assay include a specification of the average of the reference genes rather than on mass of RNA input as the primary specification for sample quality. This, in turn, allows for the processing of variable RNA inputs, and samples containing as little as 5 ng of RNA have generated reproducible results as demonstrated by the reproducibility of the individual genes and the GPS (Table 6). Volumes of the reverse transcription and qPCR reactions were reduced to preserve the genetic material, and robotic liquid handling was optimized for smaller volumes. A new genomic DNA detection assay targeting the promoter region of one of the reference genes was created, and gDNA assessment performed as part of the final qPCR step. Multiple positive and negative assay controls are included in every assay run to ensure consistent performing of the analytical process.

The estimates of analytical precision and reproducibility were obtained by analyzing 10 prostate tumor RNA samples on multiple instruments, using multiple reagent lots, by various operators over a period of 2 weeks. Standard deviation for precision for the analytical process was at or below 0.21 Cp for all gene assays and 1.86 GPS units on a 100 unit scale. Reproducibility of the analytical process, in addition to the previously mentioned sources of analytical variability, incorporated variation in RNA input levels (i.e. 5 ng, 10 ng and 20 ng inputs). Reproducibility standard deviation for the analytical process of the individual gene assays was at or below 0.21 Cp, and for the overall score was 2.11 GPS units. Given that the estimated total standard deviation in the GPS (including all pre-analytical, tissue related and biological between patient variability) is approximately 11.4, the analytical assay variability is estimated to account for only 3.4% (= 100 × 2.11^2^ / 11.4^2^) of the total variation. In a separate study (*E. Klein, manuscript submitted*), within-block reproducibility of the GPS was assessed in biopsies from 46 patients (up to 4 separate samples per biopsy-containing block). The within block standard deviation for GPS in that study was 2.8 GPS units (95% CI, 2.5 to 3.1) indicating excellent reproducibility even when histological variability is introduced.

Average amplification efficiency of the 17 gene assays in the Onco*type* DX Prostate Cancer Assay was 93%, and all gene assays were within ±6% of that value. High analytical sensitivity, wide linear range (at least 10 logs) and low bias (under 9.7%) demonstrate that the assay is able to measure accurately gene expression on a wide population range using a very limited amount of RNA.

The Onco*type* DX Prostate Cancer Assay has been clinically validated [19], demonstrating that the GPS, assessed in diagnostic biopsy tissue, can predict the likelihood of the presence of adverse pathology (high-grade and/or high-stage disease), and that it complements existing pre-treatment risk assessment tools such as PSA levels, Gleason Score, and clinical stage. The assay is intended to help guide treatment decisions in early-stage prostate cancer, including the decision between immediate therapy and active surveillance. As evidence that the analytical assay was designed well for its intended use to test RNA from small biopsies, in a clinical validation study, valid GPS results were generated for more than 95% of samples requiring 1 mm and 30 microns of tumor tissue [19].

## Conclusion

Optimization of the Onco*type* DX platform has enabled the development and analytical validation of the Onco*type* DX Prostate Cancer Assay for use with prostate biopsy specimens. This RT-PCR assay has been clinically validated to predict the risk of high grade and/or non-organ confined disease at radical prostatectomy using biopsy samples containing as little as 1 mm of tumor tissue. The Onco*type* DX Prostate Cancer Assay complements traditional clinical and pathologic diagnostic features and will assist clinicians to discriminate patients with indolent prostate cancer from aggressive prostate cancer to help make the most appropriate treatment decisions.

## Competing interests

DK, ADG, NN, DBC, KCL, JS, DW and CQ are employees and stock holders of Genomic Health Inc. EAK, CMG and SMF have received research funding from Genomic Health Inc. Patents relating to the content of this manuscript have been applied for by Genomic Health Inc. Genomic Health Inc. funded this project.

## Authors’ contributions

DK led the study and drafted the manuscript. DBC was responsible for assay designs and discussion; JS, DK, KCL, AG and CQ were responsible for study design; CQ and DW were responsible for statistical analysis. NN coordinated and performed laboratory procedures. CM-G, SMF and EAK were responsible for selection and preparation of diagnostic biopsies and data review. All authors read and approved the final manuscript.

## Supplementary Material

Additional file 1 Oligonucleotide Sequences for each primer and probe. Click here for file

Additional file 2 **Amplicon Sequences for 17 genes in the Onco** ***type *** **DX GPS.** Click here for file

## References

[B1] Siegel R Naishadham D Jemal A Cancer statistics, 2012 CA Cancer J Clin 2012 14 1 10 29 10.3322/caac.20138 22237781

[B2] Cooperberg MR Broering JM Carroll PR Time trends and local variation in primary treatment of localized prostate cancer J Clin Oncol 2010 14 7 1117 1123 10.1200/JCO.2009.26.0133 20124165 PMC2834465

[B3] Shariat SF Karakiewicz PI Roehrborn CG Kattan MW An updated catalog of prostate cancer predictive tools Cancer 2008 14 11 3075 3099 10.1002/cncr.23908 18823041

[B4] Cooperberg MR Carroll PR Klotz L Active surveillance for prostate cancer: progress and promise J Clin Oncol 2011 14 27 3669 3676 10.1200/JCO.2011.34.9738 21825257

[B5] Teutsch SM Bradley LA Palomaki GE Haddow JE Piper M Calonge N Dotson WD Douglas MP Berg AO Group EW The Evaluation of Genomic Applications in Practice and Prevention (EGAPP) Initiative: methods of the EGAPP Working Group Genet Med 2009 14 1 3 14 10.1097/GIM.0b013e318184137c 18813139 PMC2743609

[B6] Clark-Langone KM Sangli C Krishnakumar J Watson D Translating tumor biology into personalized treatment planning: analytical performance characteristics of the Oncotype DX Colon Cancer Assay BMC cancer 2010 14 691 10.1186/1471-2407-10-691 21176237 PMC3016296

[B7] Cronin M Sangli C Liu ML Pho M Dutta D Nguyen A Jeong J Wu J Langone KC Watson D Analytical validation of the Oncotype DX genomic diagnostic test for recurrence prognosis and therapeutic response prediction in node-negative, estrogen receptor-positive breast cancer Clin Chem 2007 14 6 1084 1091 10.1373/clinchem.2006.076497 17463177

[B8] Albain KS Barlow WE Shak S Hortobagyi GN Livingston RB Yeh IT Ravdin P Bugarini R Baehner FL Davidson NE Prognostic and predictive value of the 21-gene recurrence score assay in postmenopausal women with node-positive, oestrogen-receptor-positive breast cancer on chemotherapy: a retrospective analysis of a randomised trial Lancet Oncol 2010 14 1 55 65 10.1016/S1470-2045(09)70314-6 20005174 PMC3058239

[B9] Paik S Tang G Shak S Kim C Baker J Kim W Cronin M Baehner FL Watson D Bryant J Gene expression and benefit of chemotherapy in women with node-negative, estrogen receptor-positive breast cancer J Clin Oncol 2006 14 23 3726 3734 10.1200/JCO.2005.04.7985 16720680

[B10] Paik S Shak S Tang G Kim C Baker J Cronin M Baehner FL Walker MG Watson D Park T A multigene assay to predict recurrence of tamoxifen-treated, node-negative breast cancer Engl J Med 2004 14 27 2817 2826 10.1056/NEJMoa041588 15591335

[B11] Dowsett M Cuzick J Wale C Forbes J Mallon EA Salter J Quinn E Dunbier A Baum M Buzdar A Prediction of risk of distant recurrence using the 21-gene recurrence score in node-negative and node-positive postmenopausal patients with breast cancer treated with anastrozole or tamoxifen: a TransATAC study J Clin Oncol 2010 14 11 1829 1834 10.1200/JCO.2009.24.4798 20212256

[B12] Solin LJ Gray R Baehner FL Butler SM Hughes LL Yoshizawa C Cherbavaz DB Shak S Page DL Sledge GW Jr A multigene expression assay to predict local recurrence risk for ductal carcinoma in situ of the breast J Natl Cancer Inst 2013 14 10 701 710 10.1093/jnci/djt067 23641039 PMC3653823

[B13] Gray RG Quirke P Handley K Lopatin M Magill L Baehner FL Beaumont C Clark-Langone KM Yoshizawa CN Lee M Validation study of a quantitative multigene reverse transcriptase-polymerase chain reaction assay for assessment of recurrence risk in patients with stage II colon cancer J Clin Oncol 2011 14 35 4611 4619 10.1200/JCO.2010.32.8732 22067390

[B14] O'Connell MJ Lavery I Yothers G Paik S Clark-Langone KM Lopatin M Watson D Baehner FL Shak S Baker J Relationship between tumor gene expression and recurrence in four independent studies of patients with stage II/III colon cancer treated with surgery alone or surgery plus adjuvant fluorouracil plus leucovorin J Clin Oncol 2010 14 25 3937 3944 10.1200/JCO.2010.28.9538 20679606 PMC2940392

[B15] Venook AP Niedzwiecki D Lopatin M Ye X Lee M Friedman PN Frankel W Clark-Langone K Millward C Shak S Biologic Determinants of Tumor Recurrence in Stage II Colon Cancer: validation study of the 12-gene recurrence score in Cancer and Leukemia Group B (CALGB) 9581 J Clin Oncol 2013 10.1200/JCO.2012.45.1096 PMC3641698 23530100

[B16] NCCN Clinical Practice Guidelines in Oncology™: Breast Cancer (Version 2.2011) 2011 http://www.nccn.org/professionals/physician_gls/PDF/breast.pdf

[B17] Harris L Fritsche H Mennel R Norton L Ravdin P Taube S Somerfield MR Hayes DF Bast RC Jr American Society of Clinical Oncology 2007 update of recommendations for the use of tumor markers in breast cancer J Clin Oncol 2007 14 33 5287 5312 10.1200/JCO.2007.14.2364 17954709

[B18] Thorson P Vollmer RT Arcangeli C Keetch DW Humphrey PA Minimal carcinoma in prostate needle biopsy specimens: diagnostic features and radical prostatectomy follow-up Mod Pathol 1998 14 6 543 551 9647592

[B19] Cooperberg M Simko J Falzarano S Maddala T Chan J Cowan J Magi-Galluzzi C Tsiatis A Tenggara-Hunter I Knezevic D Development and validation of the biopsy-based genomic prostate score (GPS) as a predictor of high grade or extracapsular prostate cancer to improve patient selection for active surveillance J Urol 2013 14 4 e873

[B20] Epstein JI Allsbrook WC Jr Amin MB Egevad LL Committee IG The 2005 International Society of Urological Pathology (ISUP) Consensus Conference on Gleason Grading of Prostatic Carcinoma Am J Surg Pathol 2005 14 9 1228 1242 10.1097/01.pas.0000173646.99337.b1 16096414

[B21] Krouwer JS Schlain B A method to quantify deviations from assay linearity Clin Chem 1993 14 8 1689 1693 8353958

[B22] Klein EA Maddala T Millward C Cherbavaz DB Falzarano SM Knezevic D Novotny WF Lee M Magi-Galluzzi C Development of a needle biopsy-based genomic test to improve discrimination of clinically aggressive from indolent prostate cancer J Clin Oncol 2012 14 4560

[B23] Magi-Galluzzi C Millward C Maddala T Cherbavaz DB Chen A Falzarano SM Lee M Baehner FL Klein EA RNA yields and RT-PCR gene expression profiles obtained from manual-microdissected fixed paraffin-embedded prostate cancer needle core biopsies Mod Pathol 2010 14 205A 10.1038/modpathol.2009.159

[B24] Pfaffl MW A new mathematical model for relative quantification in real-time RT–PCR Nucleic Acids Res 2001 14 9 e45 10.1093/nar/29.9.e45 11328886 PMC55695

[B25] McShane LM Hayes DF Publication of tumor marker research results: the necessity for complete and transparent reporting J Clin Oncol 2012 14 34 4223 4232 10.1200/JCO.2012.42.6858 23071235 PMC3504327

