# Supplementary material for: Analytical validation of the Oncotype DX prostate cancer assay – a clinical RT-PCR assay optimized for prostate needle biopsies
Source: BMC Genomics. 2013 Oct 8;14:690. doi: 10.1186/1471-2164-14-690 (PMC4007703; doi:10.1186/1471-2164-14-690)
Supplement: Additional file 1 — Oligonucleotide Sequences for each primer and probe. [file 1471-2164-14-690-S1.docx]

| **Official**  **Symbol** | **Sequence_ID** | **Primer** | **Sequence** |
| --- | --- | --- | --- |
| ARF1 | NM_001658.2 | Forward | CAGTAGAGATCCCCGCAACT |
| ARF1 | NM_001658.2 | Reverse | ACAAGCACATGGCTATGGAA |
| ARF1 | NM_001658.2 | Probe | CTTGTCCTTGGGTCACCCTGCA |
| ATP5E | NM_006886.2 | Forward | CCGCTTTCGCTACAGCAT |
| ATP5E | NM_006886.2 | Reverse | TGGGAGTATCGGATGTAGCTG |
| ATP5E | NM_006886.2 | Probe | TCCAGCCTGTCTCCAGTAGGCCAC |
| AZGP1 | NM_001185.2 | Forward | GAGGCCAGCTAGGAAGCAA |
| AZGP1 | NM_001185.2 | Reverse | CAGGAAGGGCAGCTACTGG |
| AZGP1 | NM_001185.2 | Probe | TCTGAGATCCCACATTGCCTCCAA |
| BGN | NM_001711.3 | Forward | GAGCTCCGCAAGGATGAC |
| BGN | NM_001711.3 | Reverse | CTTGTTGTTCACCAGGACGA |
| BGN | NM_001711.3 | Probe | CAAGGGTCTCCAGCACCTCTACGC |
| TPX2 | NM_012112.2 | Forward | TCAGCTGTGAGCTGCGGATA |
| TPX2 | NM_012112.2 | Reverse | ACGGTCCTAGGTTTGAGGTTAAGA |
| TPX2 | NM_012112.2 | Probe | CAGGTCCCATTGCCGGGCG |
| CLTC | NM_004859.1 | Forward | ACCGTATGGACAGCCACAG |
| CLTC | NM_004859.1 | Reverse | TGACTACAGGATCAGCGCTTC |
| CLTC | NM_004859.1 | Probe | TCTCACATGCTGTACCCAAAGCCA |
| COL1A1 | NM_000088.2 | Forward | GTGGCCATCCAGCTGACC |
| COL1A1 | NM_000088.2 | Reverse | CAGTGGTAGGTGATGTTCTGGGA |
| COL1A1 | NM_000088.2 | Probe | TCCTGCGCCTGATGTCCACCG |
| FAM13C | NM_198215.2 | Forward | ATCTTCAAAGCGGAGAGCG |
| FAM13C | NM_198215.2 | Reverse | GCTGGATACCACATGCTCTG |
| FAM13C | NM_198215.2 | Probe | TCCTGACTTTCTCCGTGGCTCCTC |
| FLNC | NM_001458.4 | Forward | CAGGACAATGGTGATGGCT |
| FLNC | NM_001458.4 | Reverse | TGATGGTGTACTCGCCAGG |
| FLNC | NM_001458.4 | Probe | ATGTGCTGTCAGCTACCTGCCCAC |
| GPS1 | NM_004127.4 | Forward | AGTACAAGCAGGCTGCCAAG |
| GPS1 | NM_004127.4 | Reverse | GCAGCTCAGGGAAGTCACA |
| GPS1 | NM_004127.4 | Probe | CCTCCTGCTGGCTTCCTTTGATCA |
| GSN | NM_000177.1 | Forward | CTTCTGCTAAGCGGTACATCGA |
| GSN | NM_000177.1 | Reverse | GGCTCAAAGCCTTGCTTCAC |
| GSN | NM_000177.1 | Probe | ACCCAGCCAATCGGGATCGGC |
| GSTM2 | NM_000848.2 | Forward | CTGCAGGCACTCCCTGAAAT |
| GSTM2 | NM_000848.2 | Reverse | CCAAGAAACCATGGCTGCTT |
| GSTM2 | NM_000848.2 | Probe | CTGAAGCTCTACTCACAGTTTCTGGG |
| KLK2 | NM_005551.3 | Forward | AGTCTCGGATTGTGGGAGG |
| KLK2 | NM_005551.3 | Reverse | TGTACACAGCCACCTGCC |
| KLK2 | NM_005551.3 | Probe | TTGGGAATGCTTCTCACACTCCCA |
| PGK1 | NM_000291.1 | Forward | AGAGCCAGTTGCTGTAGAACTCAA |
| PGK1 | NM_000291.1 | Reverse | CTGGGCCTACACAGTCCTTCA |
| PGK1 | NM_000291.1 | Probe | TCTCTGCTGGGCAAGGATGTTCTGTTC |
| SFRP4 | NM_003014.2 | Forward | TACAGGATGAGGCTGGGC |
| SFRP4 | NM_003014.2 | Reverse | GTTGTTAGGGCAAGGGGC |
| SFRP4 | NM_003014.2 | Probe | CCTGGGACAGCCTATGTAAGGCCA |
| SRD5A2 | NM_000348.2 | Forward | GTAGGTCTCCTGGCGTTCTG |
| SRD5A2 | NM_000348.2 | Reverse | TCCCTGGAAGGGTAGGAGTAA |
| SRD5A2 | NM_000348.2 | Probe | AGACACCACTCAGAATCCCCAGGC |
| TPM2 | NM_213674.1 | Forward | AGGAGATGCAGCTGAAGGAG |
| TPM2 | NM_213674.1 | Reverse | CCACCTCTTCATATTTGCGG |
| TPM2 | NM_213674.1 | Probe | CCAAGCACATCGCTGAGGATTCAG |
|  | 1963.1 | Forward | GGGACACAGCAAGCCTCCCG |
|  | 1963.1 | Reverse | GTGGAAACCTGTGGCACTCGC |
|  | 1963.1 | Probe | CCTTCGGACCTTCCGCCGTGGCCCC |
