# Supplementary material for: Analytical validation of the Oncotype DX prostate cancer assay – a clinical RT-PCR assay optimized for prostate needle biopsies
Source: BMC Genomics. 2013 Oct 8;14:690. doi: 10.1186/1471-2164-14-690 (PMC4007703; doi:10.1186/1471-2164-14-690)
Supplement: Additional file 2 — Amplicon Sequences for 17 genes in the Onco type DX GPS. [file 1471-2164-14-690-S2.docx]

| **Official Symbol** | **Amplicon Length** | **Amplicon Sequence** |
| --- | --- | --- |
| ARF1 | 64 | CAGTAGAGATCCCCGCAACTCGCTTGTCCTTGGGTCACCCTGCATTCCATAGCCATGTGCTTGT |
| ATP5E | 66 | CCGCTTTCGCTACAGCATGGTGGCCTACTGGAGACAGGCTGGACTCAGCTACATCCGATACTCCCA |
| AZGP1 | 66 | GAGGCCAGCTAGGAAGCAAGGGTTGGAGGCAATGTGGGATCTCAGACCCAGTAGCTGCCCTTCCTG |
| BGN | 66 | GAGCTCCGCAAGGATGACTTCAAGGGTCTCCAGCACCTCTACGCCCTCGTCCTGGTGAACAACAAG |
| CLTC | 67 | ACCGTATGGACAGCCACAGCCTGGCTTTGGGTACAGCATGTGAGATGAAGCGCTGATCCTGTAGTCA |
| COL1A1 | 68 | GTGGCCATCCAGCTGACCTTCCTGCGCCTGATGTCCACCGAGGCCTCCCAGAACATCACCTACCACTG |
| FAM13C | 66 | ATCTTCAAAGCGGAGAGCGGGAGGAGCCACGGAGAAAGTCAGGAGACAGAGCATGTGGTATCCAGC |
| FLNC | 67 | CAGGACAATGGTGATGGCTCATGTGCTGTCAGCTACCTGCCCACGGAGCCTGGCGAGTACACCATCA |
| GPS1 | 66 | AGTACAAGCAGGCTGCCAAGTGCCTCCTGCTGGCTTCCTTTGATCACTGTGACTTCCCTGAGCTGC |
| GSN | 85 | CTTCTGCTAAGCGGTACATCGAGACGGACCCAGCCAATCGGGATCGGCGGACGCCCATCACCGTGGTGAAGCAAGGCTTTGAGCC |
| GSTM2 | 68 | CTGCAGGCACTCCCTGAAATGCTGAAGCTCTACTCACAGTTTCTGGGGAAGCAGCCATGGTTTCTTGG |
| KLK2 | 66 | AGTCTCGGATTGTGGGAGGCTGGGAGTGTGAGAAGCATTCCCAACCCTGGCAGGTGGCTGTGTACA |
| PGK1 | 74 | AGAGCCAGTTGCTGTAGAACTCAAATCTCTGCTGGGCAAGGATGTTCTGTTCTTGAAGGACTGTGTAGGCCCAG |
| SFRP4 | 67 | TACAGGATGAGGCTGGGCATTGCCTGGGACAGCCTATGTAAGGCCATGTGCCCCTTGCCCTAACAAC |
| SRD5A2 | 81 | GTAGGTCTCCTGGCGTTCTGCCAGCTGGCCTGGGGATTCTGAGTGGTGTCTGCTTAGAGTTTACTCCTACCCTTCCAGGGA |
| TPM2 | 66 | AGGAGATGCAGCTGAAGGAGGCCAAGCACATCGCTGAGGATTCAGACCGCAAATATGAAGAGGTGG |
| TPX2 | 65 | TCAGCTGTGAGCTGCGGATACCGCCCGGCAATGGGACCTGCTCTTAACCTCAAACCTAGGACCGT |
